# Type of anaesthesia and the safety and efficacy of thromboprophylaxis with enoxaparin or dabigatran etexilate in major orthopaedic surgery: pooled analysis of three randomized controlled trials

**DOI:** 10.1186/1477-9560-10-9

**Published:** 2012-06-18

**Authors:** Nadia Rosencher, Herbert Noack, Martin Feuring, Andreas Clemens, Richard J Friedman, Bengt I Eriksson

**Affiliations:** 1 Staff Anaesthesiologist, Department of Anaesthesiology and Intensive Care, Paris Descartes University, Cochin Hospital (AP HP), rue du Faubourg Saint-Jacques, 75014, Paris, France; 2 Medical Data Services, Boehringer Ingelheim Pharma GmbH & Co. KG, Ingelheim am Rhein, Germany; 3 Department of Clinical Development & Medical Affairs, Boehringer Ingelheim International GmbH, Ingelheim am Rhein, Germany; 4 Department of Orthopaedic Surgery, Roper Hospital, Medical University of South Carolina, Charleston, SC, USA; 5 Department of Orthopaedic Surgery, University of Gothenburg, Sahlgrenska University Hospital, Mölndal, Sweden

**Keywords:** Thromboprophylaxis, Type of anaesthesia, Venous thromboembolism, Bleeding, Dabigatran etexilate

## Abstract

**Background:**

There has been a shift towards greater use of neuraxial over general anaesthesia for patients undergoing total hip or knee arthroplasty. Furthermore, suggestions that peripheral nerve block may reduce adverse effects have recently been put forward. Although older studies showed a reduction in venous thromboembolism (VTE) with neuraxial compared with general anaesthesia, this difference has not been confirmed in studies using effective current thromboprophylaxis. We used a large data set to investigate the pattern of anaesthesia usage, and whether anaesthesia type affects efficacy and bleeding outcomes of thromboprophylaxis overall, within each treatment group, or for the novel oral anticoagulant dabigatran etexilate versus enoxaparin.

**Methods:**

Three previously reported trials compared 220 mg and 150 mg dabigatran etexilate once daily with enoxaparin after knee or hip arthroplasty. A pooled analysis was performed in patients receiving general or neuraxial anaesthesia, or the combination of either with peripheral nerve block (n = 8062). Outcome measures were major VTE plus VTE-related mortality, major bleeding and major plus clinically relevant bleeding events.

**Results:**

General, neuraxial and combination anaesthesia were used in 29%, 52% and 19% of patients, respectively. Differences in efficacy and safety between anaesthesia subgroups were small and not significant, except for a slightly higher rate of major VTE and VTE-related mortality with general versus neuraxial anaesthesia (odds ratio: 1.40; 95% confidence interval: 1.03–1.90; p = 0.035) in the overall population. There were no significant effects of anaesthesia type on efficacy or safety of dabigatran etexilate versus enoxaparin.

**Conclusions:**

Anaesthesia type did not greatly affect efficacy and safety outcomes in the pooled population of all three treatment groups. The efficacy and safety of dabigatran etexilate was comparable with enoxaparin, regardless of type of anaesthesia.

**Trial registration:**

ClinicalTrials.gov identifiers: NCT00168805, NCT00168818, NCT00152971.

## Background

Patients undergoing total hip or knee arthroplasty surgery are known to be at high risk of venous thromboembolism (VTE) [1] and hence thromboprophylaxis is recommended [2]. To date, low molecular weight heparin (LMWH) has been most frequently used to prevent VTE following orthopaedic surgery [3], but this therapy is administered subcutaneously. To optimize patient care, research into new anticoagulants has concentrated on oral therapies.

Dabigatran etexilate is a new, reversible, oral direct thrombin inhibitor that has been approved in many countries worldwide for use in orthopaedic surgery [4]. Three pivotal Phase III clinical trials – RE-MODEL™, RE-NOVATE® and RE-MOBILIZE® – investigated the efficacy and safety of 220 mg and 150 mg dabigatran etexilate once daily compared with subcutaneous enoxaparin as thromboprophylaxis in patients undergoing total hip or knee arthroplasty [5-7].

In recent years there has been a shift in the anaesthesia techniques used in orthopaedic surgery, from general to regional anaesthesia. A study by Anderson et al. shows that, in 1996, 35% of patients undergoing total hip arthroplasty and 43% undergoing total knee arthroplasty received spinal or epidural anaesthesia, while in 2001 these figures had increased to 46% and 54%, respectively [8]. In addition, peripheral nerve blocks are being increasingly used during hip and knee arthroplasty [9], and may represent the future trend.

With all anticoagulants, a major consideration is the balance between efficacy (prevention of VTE) and safety (mainly bleeding). Available evidence suggests that neuraxial anaesthesia lowered the rate of VTE compared with general anaesthesia in studies conducted before the widespread use of effective anticoagulation; but now that patients are given appropriate, risk-adjusted thromboprophylaxis with newer agents, this difference no longer exists [10]. However, a recent review of randomized controlled trials since 1990 (18 studies involving 1239 patients) concluded that there was insufficient evidence to determine whether anaesthetic technique influenced mortality, cardiovascular morbidity or the incidence of VTE when using thromboprophylaxis [11].

The following studies of dabigatran in major orthopaedic surgery provide a large data set (8062 patients): the study of thromboembolism prevention after knee surgery (RE-MODEL™), the study of extended thromboembolism prevention after hip surgery (RE-NOVATE®) and the study of thromboembolism prevention after knee surgery (RE-MOBILIZE®). Here we report the pattern of anaesthesia usage and the results of three post-hoc analyses to determine (a) whether the type of anaesthesia affects efficacy and safety outcomes in the context of contemporary patient management and thromboprophylaxis modalities in the overall study population, regardless of treatment assignment, (b) whether there was any impact of anaesthetic technique on the efficacy and safety of dabigatran relative to enoxaparin and (c) whether anaesthetic technique influenced efficacy and safety within each treatment group.

## Methods

### Trial designs

All trials were performed in compliance with the Declaration of Helsinki (1996) and the International Conference on Harmonisation (ICH) Harmonised Tripartite Guideline for Good Clinical Practice and in accordance with applicable local and national regulatory requirements. All patients provided written informed consent. The clinical trial protocols were reviewed by the ethics committees/institutional review boards of the coordinating investigators and all principal investigators of the participating centres. The EudraCT numbers were as follows: for RE-MODEL™, 2004-001317-34 (17 June 2004); for RE-NOVATE®, 2004-001988-21 (20 July 2004) for RE-MOBILIZE®, 2005-001998-10 (28 April 2005).

Full details of the three prospective, double-blind, double-dummy, randomized, multicentre, non-inferiority, pivotal trials have been reported previously [5-7]. Briefly, the European trials, RE-MODEL™ (NCT00168805) and RE-NOVATE® (NCT00168818), compared 220 mg and 150 mg once daily dabigatran etexilate (Boehringer Ingelheim International GmbH, Ingelheim am Rhein, Germany) with 40 mg once daily subcutaneous enoxaparin (Clexane, Klexane or Lovenox; Sanofi-Aventis, Paris, France), following knee or hip arthroplasty, respectively. Patients aged over 18 years were randomized the day before surgery using a computer-generated scheme. As these were double-blind trials, all patients received two oral capsules in the morning and a subcutaneous injection in the evening. The injection regime was generally initiated the evening before surgery, although in some countries it was initiated post-operatively. Dabigatran etexilate treatment was initiated as a half-dose 1–4 hours after the completion of surgery, followed by the full dose the next day. Patients were treated for 6–10 days in the RE-MODEL™ trial and for 28–35 days in RE-NOVATE®. All patients were to undergo bilateral venography, which was assessed centrally by the independent adjudication committee, within 24 hours of the last dose [5,6].

The RE-MOBILIZE® trial (NCT00152971) assessed patients undergoing knee arthroplasty. To reflect North American practice, patients were randomized after surgery to 220 mg or 150 mg dabigatran etexilate once daily, or 30 mg subcutaneous enoxaparin twice daily. Patients received oral capsules and an injection in the morning, followed by a second injection in the evening. The first injection was given 12–24 hours after surgery while dabigatran etexilate was initiated as a half dose 6–12 hours after surgery. Patients received treatment for 12–15 days and were to undergo bilateral venography (which was assessed centrally) within 12 hours of the last dose [7]. In each trial, an independent expert adjudication committee assessed the outcomes.

The three trials were designed to be as similar as possible to enable the prospectively defined meta-analyses. Thromboprophylaxis could be continued after termination of the study at the discretion of the treating physician and all patients attended a follow-up visit 3 months after surgery. Concomitant treatment with aspirin at doses <160 mg and with selective cyclooxygenase-2 inhibitors was permitted, as was the use of elastic compression stockings; use of intermittent pneumatic compression devices was not permitted. In addition, the main exclusion criteria in all three trials excluded patients with a history of bleeding diatheses, a coagulation disorder, creatinine clearance <30 mL/min, or who had undergone ≥3 attempts to place spinal or epidural anaesthesia or in whom the placement was traumatic.

### Outcome measures

The primary efficacy outcome in all trials was total VTE (symptomatic or venographic deep vein thrombosis [DVT], and/or symptomatic pulmonary embolism) and all-cause mortality, as agreed with the authorities during the planning process for the studies. However, this endpoint was not suitable for the post-hoc analysis due to the heterogeneity of the trials resulting from differences in the type of operation (hip versus knee arthroplasty), enoxaparin regimen used, timing of initiation of therapy and the duration of therapy. Hence the efficacy endpoint used in these pooled analyses was the composite of major VTE (venographic or symptomatic proximal DVT and/or pulmonary embolism) and VTE-related mortality during treatment. This was a secondary endpoint in the individual studies due to limitations regarding power and sample size. However, it was pre-specified for the meta-analyses, is more clinically relevant than total VTE and all-cause mortality, and is currently recommended by the European regulatory authorities as the primary endpoint for therapeutic non-inferiority of VTE prevention [12]. Ventilation-perfusion scintigraphy, pulmonary angiography, spiral chest computer tomography or autopsy determined the presence of pulmonary embolism. Compression ultrasound or venography were used to confirm symptomatic DVT and were assessed centrally. Deaths were considered to be due to VTE if they were categorized as VTE-related or unexplained.

Safety outcomes concentrated on bleeding events and included major bleeding events (MBE), incorporating surgical-site bleeds, and clinically relevant (non-major) bleeding events (CRBE). All bleeding events were classified as described previously [5-7,13].

### Type of anaesthesia

In these studies, general anaesthesia, spinal and/or epidural (neuraxial) anaesthesia and peripheral nerve block were used. For the post-hoc analyses, patients were classified as having received general or neuraxial anaesthesia or a combination; the combination was defined as general or neuraxial anaesthesia plus peripheral nerve block. The type of anaesthesia used was recorded in the electronic case record form.

In patients who received neuraxial anaesthesia, the recommendation was not to initiate the first dose of oral therapy for a minimum of 2 hours after the catheter was removed. Trial medication was stopped if patients had an indwelling epidural catheter for pain relief post-surgery [5-7].

### Statistics

Subgroup analyses of the pooled data from the three trials were performed to determine: (a) if the anaesthesia technique used affected the study outcomes in the overall population across all treatment groups (without looking into the different prophylactic therapies); and (b) if the different anaesthesia techniques had an influence on the efficacy and safety of 220 mg and 150 mg dabigatran etexilate compared with enoxaparin.

The efficacy analysis was based on a modified intention-to-treat population: all randomized patients who were operated on received at least one dose of study drug, and had evaluable adjudicated data on major VTE or confirmed VTE-related death during treatment. No imputation for missing data was made in any of the analyses. The safety population comprised all randomized patients who received at least one dose of study treatment (either subcutaneous injection or oral drug).

Event rates are expressed as numbers and percentages. Anaesthesia types were compared by generating odds ratios (OR; with 95% confidence intervals [CI]) in the pooled population. For comparisons between dabigatran etexilate and enoxaparin common OR were generated using a fixed effects model and the 95% CI is given for the total population in the absence of heterogeneity. In this post-hoc explorative analysis, no correction for multiplicity was applied, therefore p-values should be interpreted with caution. SAS® (version 8.2, SAS Institute Inc., Cary, NC, USA) was used to perform the statistical calculations.

## Results

**Figure 1:**
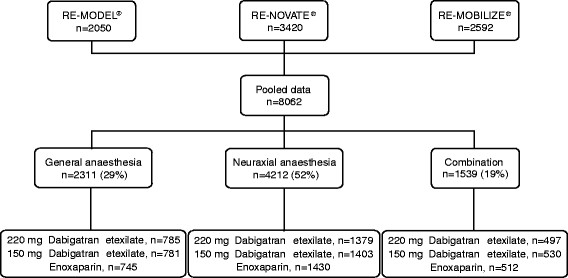
Flow diagram showing patients comprising the pooled safety population in this post-hoc analysis.

The pooled safety population comprised 8062 patients. Of these, 2311 patients (29%) received general anaesthesia, 4212 (52%) received neuraxial anaesthesia and 1539 (19%) received a combination (Figure 1). No neuraxial haematoma was observed in the 4212 patients receiving neuraxial anaesthesia, irrespective of treatment group.

### Effect of anaesthesia type in the overall population

The baseline characteristics of patients in each anaesthesia subgroup in the overall population (dabigatran and enoxaparin) were generally similar (Table 1). There were minor differences in mean age, body mass index (BMI) and creatinine clearance (CrCl) and in the proportions of patients with a history of hypertension or smoking (numerically lower in patients receiving neuraxial anaesthesia) and in those aged >75 years or with creatinine clearance <50 mL/min (numerically lower in the general anaesthesia group) but given the large size of the cohorts compared, even minor differences were statistically significant.

**Table 1:** Baseline characteristics for the pooled study population by anaesthesia subgroups

	**General anaesthesia**	**Neuraxial anaesthesia**	**Combination**
Number of patients	2311	4212	1539
Female, n (%)	1406 (60.8)	2471 (58.7)	902 (58.6)
Mean age, years ± SD	64.4 ± 10.5	66.2 ± 9.7*	65.6 ± 10.2*
Mean BMI, kg/m^2^ ± SD	30.2 ± 5.9	28.8 ± 4.9*	30.1 ± 5.8
CrCl, mL/min			
Median	86.3	82.4*	82.9*
Minimum	22.0	20.9	25.0
Maximum	295.7	289.6	231.6
Smoker or ex-smoker, n (%)	945 (40.9)	1483 (35.2)*	650 (42.2)
Hypertension, n (%)	1353 (58.5)	2203 (52.3)*	862 (56.0)
>75 years, n (%)	315 (13.6)	746 (17.7)*	266 (17.3)*
<50 CrCl, n (%)	156 (6.8)	341 (8.1)	126 (8.2)
>75 years or <50 CrCl, n (%)	371 (16.1)	870 (20.7)*	299 (19.4)*

*SD*, standard deviation; *BMI*, body mass index; *CrCl*, creatinine clearance.

*p < 0.05 when compared to general anaesthesia treatment arm.

The event rates observed with the different anaesthesia types and OR for comparisons between groups are shown in Table 2. The differences in efficacy and safety outcomes between anaesthesia subgroups in the overall population were small and not statistically significant, except for a slightly higher rate of major VTE and VTE-related mortality with general anaesthesia alone versus neuraxial anaesthesia alone (OR 1.40, 95% CI 1.03–1.90, p = 0.035).

**Table 2:** Effect of type of anaesthesia on outcomes: event rates observed with the different types of anaesthesia in the overall study population

**Outcome**	**General anaesthesia**	**Neuraxial anaesthesia**	**Combination**
Major VTE and VTE-related mortality	4.2%*	3.1%*	3.1%
	(73/1731)	(101/3305)	(35/1147)
MBE	1.2%	1.4%	1.2%
	(27/2311)	(59/4212)	(19/1539)
MBE/CRBE	4.9%	5.6%	5.7%
	(114/2311)	(237/4212)	(88/1539)

*Only significantly different odds ratio (general versus neuraxial): 1.40 (95% confidence interval: 1.03–1.90, p = 0.035); all other comparisons are not significant.

*VTE*, venous thromboembolism; *MBE*, major bleeding event; *CRBE*, clinically relevant bleeding events.

### Effect of anaesthesia type on the comparison between dabigatran and enoxaparin

**Figure 2:**
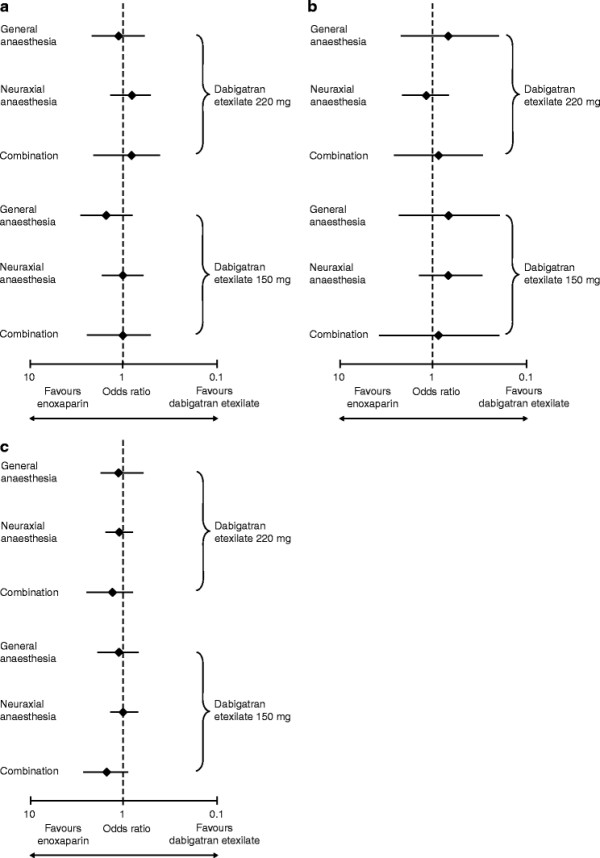
**Comparison of 220 mg and 150 mg dabigatran etexilate with enoxaparin treatments for the three outcomes analysed according to the type of anaesthesia: (A) major venous thromboembolism (VTE) and VTE-related mortality; (B) major bleeding events; (C) major bleeding events plus clinically relevant bleeding events.** Data are presented as the odds ratio with 95% confidence intervals.

Statistical comparisons of the efficacy and safety of dabigatran versus enoxaparin (OR with 95% CI) are given in Figure 2. The absolute event rates observed with 220 mg dabigatran etexilate, 150 mg dabigatran etexilate and enoxaparin for the different types of anaesthesia are also shown in Table 3. For the efficacy outcome and both safety outcomes, 220 mg dabigatran etexilate was comparable to enoxaparin (OR ranged from 0.7 to 1.3 with all 95% CIs crossing 1.0, showing no statistically significant difference), regardless of the type of anaesthesia used during surgery. Similarly, the type of anaesthesia used during surgery did not affect the efficacy and safety of 150 mg dabigatran etexilate compared to enoxaparin (OR range: 0.7–1.5 with all 95% CIs crossing 1.0, showing no statistically significant difference).

**Table 3:** Effect of type of anaesthesia on the efficacy and safety of dabigatran etexilate: event rates observed with the different types of anaesthesia for each therapy

**Outcome**	**General anaesthesia**	**Neuraxial anaesthesia**	**Combination**
Dabigatran etexilate 220 mg			
Major VTE and VTE-related mortality	4.0% (23/578)	2.7% (29/1080)	2.7% (10/369)
OR (95% CI) versus general anaesthesia		0.67 (0.37–1.22)	0.67 (0.28–1.49)
MBE	0.9% (7/785)	1.7% (24/1379)	1.4% (7/497)
OR (95% CI) versus general anaesthesia		1.97 (0.82–5.43)	1.59 (0.47–5.34)
MBE/CRBE	4.8% (38/785)	5.9% (81/1379)	6.0% (30/497)
OR (95% CI) versus general anaesthesia		1.23 (0.82–1.87)	1.26 (0.74–2.12)
Dabigatran etexilate 150 mg			
Major VTE and VTE-related mortality	5.1% (30/587)	3.2% (35/1082)	3.3% (13/395)
OR (95% CI) versus general anaesthesia		0.62 (0.37–1.06)	0.63 (0.30–1.27)
MBE	1.3% (10/781)	1.0% (14/1403)	0.8% (4/530)
OR (95% CI) versus general anaesthesia		0.78 (0.32–1.97)	0.59 (0.13–2.05)
MBE/CRBE	5.4% (42/781)	5.4% (76/1403)	6.6% (35/530)
OR (95% CI) versus general anaesthesia		1.01 (0.67–1.52)	1.24 (0.76–2.03)
Enoxaparin			
Major VTE and VTE-related mortality	3.5% (20/566)	3.2% (37/1143)	3.1% (12/383)
OR (95% CI) versus general anaesthesia		0.91 (0.51–1.68)	0.88 (0.39–1.92)
MBE	1.3% (10/745)	1.5% (21/1430)	1.6% (8/512)
OR (95% CI) versus general anaesthesia		1.10 (0.49–2.62)	1.17 (0.40–3.31)
MBE/CRBE	4.6% (34/745)	5.6% (80/1430)	4.5% (23/512)
OR (95% CI) versus general anaesthesia		1.24 (0.81–1.93)	0.98 (0.55–1.74)

No statistically significant differences were observed between the dabigatran etexilate treatment groups and enoxaparin.

*VTE*, venous thromboembolism; *OR*, odds ratio; *CI*, confidence interval; *MBE*, major bleeding event; *CRBE*, clinically relevant bleeding event.

### Effect of anaesthesia type in the individual treatment groups

Table 3 includes the statistical comparison of event rates in the neuraxial anaesthesia or combination anaesthesia subgroups versus the general anaesthesia subgroup within each treatment. There was no clear pattern in the effect of anaesthesia type on rates of MBE; in fact, any potential trends were reversed between the 220 mg and 150 mg dabigatran groups. Event rates appeared similar across anaesthesia subgroups in the enoxaparin arm.

## Discussion

In these studies the majority of patients received neuraxial anaesthesia, which is consistent with the trend in clinical practice in Europe observed in the early 2000s [8]. Compared with systemic analgesia, regional analgesia can reduce post-operative pain, morphine consumption, and nausea and vomiting; it may also reduce blood loss after total hip arthroplasty [11]. In a more recent trend, lower limb blocks are increasingly used for analgesia for total joint arthroplasty and other surgeries, in either a single-shot or continuous technique. These provide effective analgesia and may be associated with fewer adverse effects and potentially earlier mobilization of patients [9,14,15]. It is perhaps surprising, therefore, that only 19% of patients in our large patient population (two European-based studies and one North American based) received a combination of general or neuraxial anaesthesia with a peripheral nerve block.

In our analysis of outcomes according to anaesthesia type in the overall study population of more than 8000 patients (dabigatran and enoxaparin groups combined), patients receiving general anaesthesia alone had a higher rate of major thromboembolic events compared with neuraxial anaesthesia alone, which just reached significance (1.4-fold, 1.1% absolute risk difference; OR 1.40, 95% CI 1.03–1.90, p = 0.035); that is, patients receiving neuraxial anaesthesia benefited from slightly lower rates of VTE. However, there was no significant difference between general anaesthesia alone and the combination. Due to the post-hoc nature of these analyses and the multiplicity arising from several comparisons, findings should be interpreted with caution. With regard to safety outcomes (MBE and MBE/CRBE), there was no significant effect of anaesthesia type, despite the pooled groups being large enough and there being adequate power to find relevant differences. Other studies of the newer anticoagulants found no difference between the rate of DVT with general and neuraxial anaesthesia [16,17]. While a reduction in the risk of morbidity and mortality (including VTE) with neuraxial anaesthesia has been reported in several trials of general versus neuraxial anaesthesia for orthopaedic surgery [18-20], these studies were undertaken before the widespread use of thromboprophylaxis. Hence, this evidence is not corroborated by more recent studies [21]. The value of meta-analyses has been limited as they have included the older studies [22-24]. However, a recent review of randomized controlled trials since 1990 concluded that there was insufficient evidence from these trials, involving 1239 patients, to determine whether anaesthetic technique influenced mortality, cardiovascular morbidity or the incidence of VTE when using thromboprophylaxis [11].

In the pooled results of the RE-MODEL™, RE-NOVATE® and RE-MOBILIZE® trials, major VTE and VTE-related mortality occurred in 3.3% of the enoxaparin group, 3.0% of the 220 mg dabigatran etexilate group and 3.8% of the 150 mg dabigatran etexilate group; MBE occurred in 1.4%, 1.4% and 1.1%, respectively; and MBE/CRBE in 5.0%, 5.6% and 5.6%, respectively. There were no significant differences between dabigatran and enoxaparin [25]. Our comparisons of dabigatran etexilate with enoxaparin in the pooled data sets demonstrated that the 220 mg dose of dabigatran etexilate, approved for use in the majority of patients undergoing orthopaedic surgery, was as effective and safe as enoxaparin, regardless of the type of anaesthesia used during surgery. Similarly, 150 mg dabigatran etexilate showed no statistically significant differences compared with enoxaparin regardless of anaesthesia type (Figure 2 shows that all 95% CIs for the OR values all crossed 1.0).

In our comparison of the effect of anaesthesia type on results within each treatment group, we observed a possible trend towards more effective prevention of major VTE and VTE-related mortality when using either neuraxial or combination anaesthesia as opposed to general anaesthesia in the dabigatran groups. There was no clear direction of effect of anaesthesia on MBE, and event rates were similar across all anaesthesia types with enoxaparin treatment. It is important to note, however, that since this is a pooled analysis of studies that did not use stratification in randomization with respect to type of anaesthesia, the results should be interpreted with caution. Bias may have been introduced by differences in the subpopulations. Although baseline characteristics of patients in each anaesthesia subgroup generally appeared to be similar (Table 1), some differences have reached statistical significance due to the large cohort size. In addition, centres have different preferences to the type of anaesthesia and these may have confounded results.

A potential difference between using an oral compound versus a subcutaneous injected compound is the variability of oral absorption, particularly in the first 6 hours after surgery [26]. When general anaesthesia is used without regional anaesthesia, post-operative pain following this kind of major surgery has to be relieved with morphine, which can induce nausea and vomiting leading to delayed absorption of oral compounds. Use of regional anaesthesia reduces opioid consumption in the post-operative period (and thus nausea and vomiting), resulting in more normal absorption [26]. Although the recommendation is to initiate therapy with dabigatran etexilate within 1–4 hours after surgery using a half dose, if this is not possible (e.g. due to vomiting) therapy should be initiated either later the same day with a half dose or the following day using the full dose. A post-hoc subgroup analysis of data on delayed dosing versus initiation of therapy 1–4 hours post-surgery from the RE-MODEL™ and RE-NOVATE® trials (in the 220 mg dabigatran etexilate once-daily group) found that delayed initiation of therapy did not affect the efficacy and safety of dabigatran etexilate for thromboprophylaxis following orthopaedic surgery [27]. It is worth noting that the majority of delays in initiation of therapy were for logistical and other reasons, including late-day surgery, organization of the ward and nursing error rather than clinical reasons such as bleeding, drainage, catheters or vomiting [27].

With regard to MBE, it is important to note that, in the European studies, half the events seen with dabigatran etexilate had an onset before the first dose had been given (1–4 hours after surgery, according to the protocol) [5,6,28]. In the European studies enoxaparin therapy was initiated pre-surgery.

The 150 mg dabigatran etexilate dose was tested as an alternative lower dose for use in certain patient groups with an increased risk of bleeding, such as patients aged over 75 years and those with moderate renal impairment [28]. The summary of product characteristics recommends the 150 mg dose for these patients, thus thromboprophylaxis can be tailored for these more fragile individuals. Patients with pre-operative intermediate renal impairment should have their renal function monitored post-operatively, since deterioration of renal function is a risk in this patient group during the early post-operative period. Patients with severe renal impairment (creatinine clearance <30 mL/min) were excluded from the studies.

In conclusion, neuraxial anaesthesia was the most common technique, while combination with a peripheral nerve block was limited to 19% of the operations. The type of anaesthesia used during orthopaedic surgery did not greatly affect the efficacy and safety outcomes in the pooled population, although neuraxial anaesthesia appeared to be associated with a slightly lower incidence of VTE than general anaesthesia (OR 1.40). The efficacy and safety of dabigatran etexilate was comparable to enoxaparin in the overall study population and this result was maintained regardless of the type of anaesthesia. Dabigatran etexilate provides an option for thromboprophylaxis following orthopaedic surgery and has a good balance between efficacy and safety, with the advantage of oral administration and the ability to tailor the dose for elderly and renally impaired patients.

## Abbreviations

CRBE, Clinically relevant (non-major) bleeding events; CI, Confidence intervals; DVT, Deep vein thrombosis; ICH, International Conference on Harmonisation; MBE, Major bleeding events; OR, Odds ratio; VTE, Venous thromboembolism.

## Competing interests

The studies described in this paper were supported by Boehringer Ingelheim. NR, RF and BIE have been consultants or scientific advisers to, given presentations on behalf of and/or have received research funding from Boehringer Ingelheim. HN, MF and AC are employees of Boehringer Ingelheim.

## Authors’ contributions

NR, BE, MF, AC and RJ all contributed significantly to design and interpretation of the analysis and HN performed the statistical analysis. All authors contributed to the draft and revision of the manuscript and approved the final manuscript.
